# Factors affecting farmers’ use of organic and inorganic fertilizers in South Asia

**DOI:** 10.1007/s11356-021-13975-7

**Published:** 2021-05-13

**Authors:** Jeetendra Prakash Aryal, Tek Bahadur Sapkota, Timothy J. Krupnik, Dil Bahadur Rahut, Mangi Lal Jat, Clare M. Stirling

**Affiliations:** 1 International Maize and Wheat Improvement Center (CIMMYT), Carretera México-Veracruz, Km. 45, El Batán, Texcoco, Mexico; 2 International Maize and Wheat Improvement Center (CIMMYT), Dhaka, Bangladesh; 3 Asian Development Bank Institute (ADBI), Kasumigaseki Building, Level 8, 3 Chome-2-5 Kasumigaseki, Chiyoda City, Tokyo 100-6008 Japan; 4 International Maize and Wheat Improvement Center (CIMMYT), Delhi, India

**Keywords:** Inorganic fertilizer, Manure, India, Nepal, Bangladesh

## Abstract

Fertilizer, though one of the most essential inputs for increasing agricultural production, is a leading cause of nitrous oxide emissions from agriculture, contributing significantly to global warming. Therefore, understanding factors affecting farmers’ use of fertilizers is crucial to develop strategies to improve its efficient use and to minimize its negative impacts. Using data from 2528 households across the Indo-Gangetic Plains in India, Nepal, and Bangladesh, this study examines the factors affecting farmers’ use of organic and inorganic fertilizers for the two most important cereal crops – rice and wheat. Together, these crops provide the bulk of calories consumed in the region. As nitrogen (N) fertilizer is the major source of global warming and other environmental effects, we also examine the factors contributing to its overuse. We applied multiple regression models to understand the factors influencing the use of inorganic fertilizer, Heckman models to understand the likelihood and intensity of organic fertilizer (manure) use, and a probit model to examine the over-use of N fertilizer. Our results indicate that various socio-economic and geographical factors influence the use of organic and inorganic fertilizers in rice and wheat. Across the study sites, N fertilizer over-use is the highest in Haryana (India) and the lowest in Nepal. Across all locations, farmers reported a decline in manure application, concomitant with a lack of awareness of the principles of appropriate fertilizer management that can limit environmental externalities. Educational programs highlighting measures to improving nutrient-use-efficiency and reducing the negative externalities of N fertilizer over-use are proposed to address these problems.

## Introduction

Achieving food security, addressing climate change, and halting environmental and natural resource degradation are among the key challenges the agricultural sector faces in efforts to achieve sustainable development goals (SDGs^1^) and the Paris Agreement to limit the global temperature increase to below 2 °C (Wollenberg et al. 2016). Fertilizer use, particularly nitrogen (N), is an important management practice to increase crop production and improve soil fertility. Thus, the use of soil fertility enhancing amendments to supply essential nutrients in crop production is of clear importance. Along with the nutrient supply from soil organic matter, crop residues, wet and dry deposition, and biological nitrogen fixation, synthetic (inorganic) fertilizer is a primary source of essential nutrients in crop production.

The success of the Green Revolution (GR) in 1960s to increase food production and to reduce hunger worldwide was made possible, partly due to the increasing use of inorganic fertilizer (Erisman et al. 2008). However, excessive inorganic fertilizer use during and post GR caused a number of environmental and ecological problems such as soil acidification, degradation, and water eutrophication, severely undermining the sustainability of agriculture (Lu and Tian 2017). The loss of applied nutrients into the environment resulted in the fertilizer-induced emission of nitrous oxide (N_2_O) from agricultural production, a major source of anthropogenic greenhouse gas emissions (Sutton et al. 2013). Around 60% of nitrogen pollution is estimated to originate from crop production alone, particularly through nitrogen (N) fertilizer application (Sapkota et al. 2018b). Hence, agricultural development pathways need to address these concerns, in addition to climate change adaptation and mitigation (Aryal et al. 2020a; Aryal et al. 2020b; van Beek et al. 2010).

In South Asia (SA), the use of N fertilizer has been increasing over three decades (FAO 2021). Increased use of inorganic fertilizer together with irrigation and improved genetics were core to the GR philosophy that aimed to increase crop productivity in SA (Benbi 2017; Firdousi 1997; Pingali 2012; Roy 2017). Food-grain production in India increased from 82 million tons in 1960 to 284 million tons in 2018/19, rendering the country largely self-sufficient in cereals (GoI 2020). Yet to achieve this, rates of inorganic fertilizer application have increased dramatically (Benbi 2017; Roy 2017). For instance, in Indian states of Punjab and Haryana, fertilizer-N use increased from a meager 2–8 kg N ha^−1^ in 1960s to more than 160–180 kg N ha^−1^ in 2017 (Benbi 2017).

Many farmers in SA are unaware of scientifically recommended rates of fertilizer application. Rather, they apply fertilizers when and where they believe them to be necessary, often in quantities and with elements based on what are available and affordable at markets (Kishore et al. 2019; Takeshima et al. 2016). Further, recommendations for fertilizer rates tend to be based upon small-plot crop yield response data that are extrapolated over large geographic areas without considering spatial variability in the nutrient supplying capacity of the soils and temporal variability due to management factors (Ladha et al. 2020). Heavy subsidies for N fertilizer relative to other nutrients, and the lack of adequate knowledge on fertilizer management have resulted in unbalanced fertilizer application (Kishore et al. 2019). Inappropriate and unbalanced nutrient addition not only reduces nutrient use efficiency (NUE) and profitability (Krupnik et al. 2004; Ladha et al. 2005), but also increases environmental risks associated with the loss of unused nutrients through emissions, leaching or run-off (Sapkota et al. 2014). SA has one of the lowest NUE in the world (Ladha et al. 2020). Average efficiency of fertilizer N in India has been reported to be 30–40% in rice and 50–60% in other cereals (Brar et al. 2011).

Opportunities exist to improve NUE through the adoption of better fertilizer management practices such as adjusting application rates based on a more precise estimate of plant demand, using the right form of fertilizer, and using the right method to apply fertilizer so that it is delivered directly to the root zone (Dobermann and Witt 2004). Adoption of precision nutrient management technologies in India would substantially reduce fertilizer N consumption, thereby reducing GHG emission of 17.5 Mt CO2e per year with an estimated cost saving of 100 USD per t CO2e abated (Sapkota et al. 2019). Also, as the application of organic fertilizer (manures) contributes to the retention of inorganic fertilizer N and reduction of losses, mainly through gradual improvements in soil structure and ability to store nitrogen for slow release from soil organic matter (Krupnik et al. 2004; Ladha et al. 2020; Ladha et al. 2005), an understanding of the farmers’ behavior regarding the use of organic and inorganic fertilizers is crucial for designing fertilizer use policy that can address the climate change and related environmental issues in agriculture.

Realization of the benefits of improved fertilizer and organic matter management depends on the extent to which farmers are aware of and able to implement new appropriate agronomic management techniques. Farmers’ use of different management practices is difficult to predict and depends largely on the socio-economic and cultural context under which they operate (Aryal et al. 2018c; Sapkota et al. 2018a). Understanding farmer behavior toward use of inorganic and organic fertilizers is crucial because improving N fertilizer use efficiency can substantially lower the carbon footprints of agriculture (Liu et al. 2016). Though methods to improve the NUE continue to be developed, inefficient use of fertilizer persists. To date, relatively little is known about farmers’ behavior toward fertilizer and manure use in SA (Sapkota et al. 2018a; Stuart et al. 2014).

This study explores the factors affecting the use of inorganic (urea and di-ammonium phosphate) and organic (manure) fertilizers in rice and wheat production in SA, using data from 2528 households spread across Bangladesh, India, and Nepal. Urea and di-ammonium phosphate constitute over 90% of total fertilizer N applied in cropland in this region. The study also examines the factors associated with the overuse of N fertilizer (both inorganic and organic) in rice and wheat. This study contributes to the existing literatures in several ways. First, it is the first study that assesses the factors explaining farmers’ fertilizer use behavior across these countries, while controlling for farmer characteristics, socio-economic factors, and farmland characteristics. Second, as the over-use of N fertilizer is one of the major reasons behind nitrous oxide emission and other negative environmental externalities, we examine the factors explaining its over-use using both quantitative and qualitative methods. It helps us provide insights into designing low emission agricultural development and making investments consistent with the SDGs.

## Study area and data

This study used data collected from a survey of 2528 households across the Indo-Gangetic plains of Bangladesh, India, and Nepal in 2013. The data was collected through multistage sampling. In the first stage, three South Asian countries were purposively selected as they comprise approximately 84% of the land area allocated to rice and wheat in the Ingo-Gangetic Plains (Timsina and Connor 2001). In the second stage, three districts in Bangladesh (Bagerhat, Jhalokhati, and Satkhira), one district in the Terai (plain lands) region of Nepal (Rupandehi), one district in Bihar state (Vaishali), and one district in Haryana state in India (Karnal) were selected. A total of 38 villages were selected for the study: 14 from Bangladesh, 12 from Bihar (India), 13 from Haryana (India), and 12 from Nepal (Table 1).

**Table 1:** Study villages and sample size (n)

Bangladesh^1^		Bihar-India: Vaishali district		Haryana-India:Karnal district		Nepal: Rupandehi district	
Village name	n	Village name	n	Village Name	n	Village name	n
Boro Galua (J)	32	Bhatha Dasi	63	Anjanthali	67	Aahirauli	50
Burigoalini (S)	45	Bilandpur	68	Bir Narayana	49	Bairiyan	50
Chandipur (S)	66	Dedhpur	46	Barthal	41	Bhaglapur	32
Dumuria (S)	64	Dhabhaich	46	Churni Jagir	20	Dewapar	59
Durgapur (J)	8	Laxminarayanpur	44	Darar	25	Dhakdahi	92
Gabgasia (B)	66	Mirpur	55	Garghi Jattan	46	Haraiya	47
Gopalpur (J)	32	Mukundpur	69	Hathlana	46	Hati Bangai	33
Hatsala (S)	28	Panapur Camp	56	Mohri Jagir	40	Mahuwari	71
Horinagor (S)	45	Raja Pakar	70	Nanhara	43	Parasi Thuga	66
Jagannathpur (J)	64	Rampur Ratnagar	45	Pakhana	80	Razadh	36
Joka (B)	40	Rasalpour	48	Sandhir	64	Rehara	48
Sreefal Kathi (S)	45	Varishpur	31	Sanwat	45	Samrahana	47
Tarabunia (J)	45			Sounkra	60		
Teligati (B)	50						
Total sample HHs	630		641		626		631
No. of rice plots	1182		1299		665		1576
No. of wheat plots	0		1544		667		977

Note: ^1^B, J, and S denote for Bagerhat, Jhalokathi, and Satkhira districts in Bangladesh, respectively

In Bangladesh, agricultural input data were obtained from 1,182 rice fields, cultivated by 630 households. The same data were collected from 1,576 rice and 977 wheat fields operated by 631 households in Nepal, 665 rice and 667 wheat fields operated by 630 households in Haryana, and 1,299 rice and 1,604 wheat fields operated by 641 households in Bihar. Therefore, we analyze the factors affecting the use of fertilizer for rice and wheat in Nepal and India, and only for rice in Bangladesh. Furthermore, we collected qualitative information about the use of organic and inorganic fertilizers and possible reasons for their inappropriate use through two focus group discussions in each study site.

## Empirical methods

### Multiple regression models: to analyze the factors affecting urea and DAP use in rice and wheat

As all sampled farmers growing rice and wheat applied urea and DAP, we used multiple regression models to analyze the factors affecting the use of these fertilizers in each crop. Farmers’ use of inorganic fertilizers can be affected by several factors, including household characteristics, socio-economic variables, market access, and information, and farm characteristics, including soil fertility status, irrigation, and soil depth (Fishman et al. 2016; Kpadonou et al. 2019; Shrestha et al. 2013; Ward et al. 2019). We included a country dummy to capture the differences in fertilizer subsidy policy and price controls. Therefore, the following empirical model is estimated:

1 $$ {Y}_i=\alpha +\beta {X}_h+\gamma {X}_s+\delta {X}_k+\psi {X}_f+\varepsilon \kern0.36em $$

where *Y*_*i*_ denotes the amount of urea (DAP) applied per hectare (kg ha^−1^) of rice or wheat produced per season, *X*_*h*_ is a matrix of household characteristics including age, education, and gender of the household head, *X*_*s*_ is a matrix of the ownership of and access to economic resources, *X*_*k*_ represents a matrix of knowledge enhancing activities such as participation in agricultural trainings and access to extension services, in addition to access to and market information and markets, and *X*_*f*_ refers to biophysical and farm characteristics including soil depth, soil fertility, access to irrigation, and distance from homestead to the rice or wheat field. *α*, *β*, *γ*, *δ*_,_ and *ψ* are unknown parameters to be estimated, and *ε* is the stochastic error term. We controlled for possible heteroskedasticity using the Huber–White robustness test and checked for multi-collinearity using variance influence factor (Wooldridge 2010).

### Heckman two-step model: to analyze the factors affecting the application of manures to rice and wheat fields

Considering that many rice and wheat fields did not receive manure, we applied the Heckman two-step model to acknowledge censoring in the data (Heckman 1979). The Heckman model consists of two sequential decisions: first, a household decides whether or not to apply manure, and second, if they do apply it, they also need to decide how much to apply. Hence in the first step, we estimate a probit model with a dichotomous dependent variable (0 if manure is not applied and 1 if it is applied). In the second step, we analyze the factors influencing the quantity of manure used. Given that farmers applying manure have made the decision to use manure, we base this analysis on a sub-set of the data. As such, we assume that unobserved factors, which differentiate users from non-users, can also influence the amount of manure applied, resulting in selection bias. Consequently, we control for the self-selectivity bias described by using Inverse Mills Ratio (Heckman 1979). The first step (selection mechanism) in a Heckman two-step model is:

2 $$ {z}^{\ast }=\gamma w+\upsilon $$

As *z*^∗^ is not directly observable, we assume idiosyncratic criteria are applied by farmers. *z*^∗^ may be the difference in expected returns between applying and not applying manure. Therefore, a binary variable *z* is defined, which takes on the value of one if the household decides to apply manure and zero otherwise.

2a $$ z=\gamma w+\upsilon, \kern0.48em \mathrm{where}\ z=1\kern0.24em \mathrm{if}\ {\mathrm{z}}^{\ast }>0\;\mathrm{and}\ z=0\;\mathrm{otherwise}. $$

Therefore, Prob(*z* = 1) = Prob(z^∗^ > 0 ) = Prob(*υ* >  ‐ *γw*) = Φ(*γw*), where Φ is the cumulative distribution at *γw*.

The second step in the Heckman model is given by:

3 $$ y=\beta x+\varepsilon $$

Equation 3 is observed only if *z*^∗^ > 0. When *υ* and *ε* follow a bivariate normal distribution with mean of zero, standard deviation *σ* and correlation *ρ*, we get:

4 $$ E\left[y\left|z=1\right.\right]=E\left[y\left|{z}^{\ast }>0\right.\right]=\beta x+\rho \sigma \lambda \left(\gamma w\right) $$

where *λ* is the Inverse Mills Ratio (IMR) [*λ* = *ϕ*(*γw*)/Φ(*γw*)] i.e., the ratio of the value of density function of a standard normal distribution and cumulative distribution function calculated at *γw*. In Equation 4, *ρσ* is equal to the regression coefficient on the IMR, *β*_*λ*_.inclusion of IMR in the second stage as an explanatory variable helps correct selection bias. After this, the ordinary least-square method can be used for estimation as follows (see Wooldridge 2010):

5 $$ y=\beta x+{\beta}_{\lambda}\lambda +\varepsilon $$

where *β*_*λ*_ is the coefficient of the IMR. Statistically significant IMR implies selection bias and confirms the appropriateness of Heckman’s two-step model, supporting the hypothesis that the set of variables influencing the likelihood to adopt can be different from variables affecting the intensity of its use.

### Probit model: to analyze factors influencing the over-use of N fertilizer

We applied probit model to examine the factors associated with the over-use of N in rice and wheat. To obtain the total amount of N applied, we take the sum of nitrogen from urea, DAP, and manure, accounting for their standard nutrient composition at 46%, 18%, and 0.5%, respectively (Bishwakarma et al. 2015; IFFCO 2021a, b; Khatun and MA 2016). For defining the over-application of N, we followed the findings from (Sapkota et al. 2018a) in Bihar and Haryana in India and adapted it for our study sites. According to their study, wheat farmers who applied more than 140 kg N ha^−1^ suffered a yield penalty and had high greenhouse gas emission intensity. Similarly, rice yield leveled off with increased N application beyond 100 kg N ha^−1^. Based on Sapkota et al. (2018a), we created two variables, (1) “over-use of N for rice” and (2) “over-use of N for wheat”. The variable first is a binary variable with a value of one if the amount of nitrogen applied is more than 120 kg N ha^−1^ in rice fields, and zero otherwise. Similarly, the variable “over-use of N for wheat” is a binary variable with a value of one if the amount of nitrogen applied is more than 140 kg N ha^−1^ in wheat plots and zero otherwise. Hence, we applied probit model combining all locations together (for details, see Wooldridge 2010).

### Variables and hypotheses

We estimated eight econometric models each with the following dependent variable: (a) urea applied (kg per hectare) to rice, (b) urea applied (kg per hectare) to wheat, (c) DAP applied (kg per hectare) to rice, (d) DAP applied (kg per hectare) to wheat, (e) decision to use manure to rice, (f) manure applied (kg per hectare) to rice, (g) decision to use manure to wheat, and (h) manure applied (kg per hectare) to rice.

#### Explanatory variables

Based on the theoretical framework and the previous literature on technology adoption, we included explanatory variables in the empirical analysis (Aryal et al. 2018b; Aryal et al. 2018c; Pingali et al. 2019; Takeshima et al. 2016; Ward et al. 2019). Description of explanatory variables and hypotheses about their effects is summarized below.

##### Household characteristics

Household characteristics including education, age, and gender of household head, family size, and migration can influence technology adoption decisions. Educated individuals are assumed to be able to acquire new information more easily and are more likely to adopt technology (Chowdhury et al. 2014). Past studies indicate that educated individuals are more likely to use chemical fertilizer (Omamo et al. 2002; Takeshima et al. 2016). Further, elderly farmers apply more fertilizer relative to manure, as its use requires less labor compared to manure application. Conversely, with longer experience in agricultural management and the benefits of organic matter in improving soil fertility, older farmers have also been observed to prefer manure (Waithaka et al. 2007). Rural out-migration reduces the availability of household members to perform farm tasks; however, it also increases access to alternative income streams through remittances that can assist in purchasing fertilizer.

##### Economic and social capital

Economic capital consists of land, livestock, farm assets, household endowments, and off-farm income sources, whereas social capital can include membership in village institutions such as farmer cooperatives/clubs. To capture the effect of wealth on the use of fertilizer and manure, we constructed a household asset index (AI) using principal component analysis (for details, see https://www.stata.com/manuals13/mvpca.pdf). Wealthier households with higher asset index values are hypothesized to use more fertilizer (Omamo et al. 2002; Waithaka et al. 2007). By alleviating cash constraints, access to off-farm income and remittances was also hypothesized to facilitate the use of fertilizer (Pingali et al. 2019).

##### Market, institutional services, and training

Access to markets and institutional services can influence transaction costs and the degree of farmers’ knowledge and access to information, thereby influencing technology adoption (Aryal et al. 2018a; c; Chowdhury et al. 2014). Though extension staff is mobile, lack of sufficient staff to cover all the geographical territory is common in SA. We, therefore, consider distances to village markets and to extension service offices as proxies for accesses to markets and information services, respectively. Households farther from input markets conversely tend to use less fertilizer (Zhou et al. 2010). Farmers’ participation in agriculture-related trainings and educational programs also influences technology adoption (Aryal et al. 2018c).

##### Farmland characteristics

To control for the potential effects of land attributes on fertilizer/manure use, we included farmers’ tenure status, soil fertility, soil depth, land slope, irrigation status, and distance to plot from a homestead in the analysis. Distant plots require increased transaction costs due to the price of purchasing transport for inputs. Fields far from the household are also more difficult to monitor. Therefore, input use was hypothesized to be inversely related to distance from the household to the field. Farmers want to supply adequate inputs to fields that they can more closely monitor and intervene with management to achieve greater productivity. Fields that farmers perceive as less fertile may also receive more manure than fertile ones because manure releases nutrients slowly and can improve soil physical and chemical properties over time (Waithaka et al. 2007).

## Results and discussion

### Descriptive statistics

Considerable differences were observed in the application of urea and DAP across study sites (Table 2). The average amount of urea applied to rice was 315 kg ha^−1^ in Haryana (India), while it is only 205 kg ha^−1^ in Nepal. The average amount of DAP applied in rice was highest in Haryana (130 kg ha^−1^), followed by Bihar (95 kg ha^−1^), Bangladesh (65 kg ha^−1^), and then Nepal (62 kg ha^−1^). The average amount of urea applied in rice is much higher in Bangladesh compared to Bihar and Nepal. Generally, average rates of urea and DAP applied in both rice and wheat were lowest in study sites in Nepal when compared with India and Bangladesh.

**Table 2:** Description of variables used in the study

Variables	Haryana		Bihar		Bangladesh		Nepal		Overall		Variable Description
	Mean	S.D	Mean	S.D	Mean	S.D.	Mean	S.D.	Mean	S.D.	
Dependent variables											
Urea_rice (C)	315	93	210	87	285	101	205	91	254	93	Amount of urea applied in rice (kg ha^−1^)
Urea_wheat (C)	320	96	225	79	na	na	210	73	252	82	Amount of urea applied in wheat (kg ha^−1^)
DAP_rice (C)	130	49	95	53	65	29	62	45	88	44	Amount of DAP applied in rice (kg ha^−1^)
DAP_wheat (C)	125	35	110	51	na	na	79	43	104	43	Amount of DAP applied in wheat (kg ha^−1^)
Manure_rice (C)	1899	1409	925	1586	1120	950	1362	1102	1326	1261	Amount of manure applied in rice (kg ha^−1^)
Manure_wheat (C)	1680	1233	1250	955	na	na	1030	725	1320	971	Amount of manure applied in rice (kg ha^−1^)
Manure_r (D)	0.46	0.49	0.47	0.50	0.26	0.36	0.47	0.49	0.42	0.45	1 if manure is applied in rice plots
Manure_w (D)	0.25	0.29	0.43	0.48	na	na	0.24	0.34	0.31	0.36	1 if manure is applied in wheat plots
Independent variables											
Household (HH) characteristics											
Male headed HH (D)	0.97	0.17	0.91	0.29	0.90	0.30	0.78	0.41	0.89	0.31	1 if male headed house and 0 if female
Age of HH head (C)	49	13	51	14	47	13	50	14	49	13.45	Age of household in years
Education of HH head (D)	0.67	0.47	0.62	0.49	0.71	0.45	0.51	0.5	0.63	0.48	1 if HH went to school and 0 otherwise
Illiterate	0.22	0.41	0.38	0.49	0.29	0.45	0.48	0.50	0.22	0.41	Illiterate
Primary education	0.19	0.39	0.09	0.29	0.33	0.47	0.19	0.38	0.33	0.47	Up to primary education (Grades 1–5)
Secondary education	0.40	0.49	0.38	0.48	0.31	0.46	0.28	0.45	0.34	0.48	Up to secondary education (Grades 6–10)
Higher education	0.18	0.38	0.15	0.36	0.07	0.26	0.05	0.22	0.12	0.32	Higher secondary or above education (Grade 11 and above)
Education of Spouse (D)	0.51	0.5	0.3	0.46	0.63	0.48	0.22	0.41	0.42	0.49	1 if HH's spouse went to school and 0 otherwise
AEC (C)	4.46	1.79	0.89	0.17	3.27	1.27	4.57	2.19	3.28	2.15	Household labor worked in agriculture
Family Size (C)	6.03	2.47	6.05	2.65	4.67	1.68	6.39	3.08	5.78	2.61	Total members in family
Food Security status (D)	1	0.07	0.69	0.46	0.72	0.45	0.89	0.3	0.82	0.37	1 if HH is food secure and 0 otherwise
Migration (D)	0.11	0.31	0.28	0.45	0.23	0.42	0.38	0.48	0.25	0.43	1 if HH has migrant member and 0 otherwise
Economic and social capital											
Land size (C)	3.33	3.81	0.51	0.41	0.44	0.48	0.49	0.85	1.1	2.32	Total land operated in ha
TLU (C)	3.81	5.7	0.70	0.74	0.92	1.19	1.25	1.54	1.63	3.27	Livestock owned in tropical livestock unit
Asset index (C)	0.93	0.61	0.30	0.53	0.43	0.75	0.45	0.68	0.53	0.59	Household asset index ^1^
Access to credit (D)	0.4	0.49	0.34	0.48	0.69	0.46	0.44	0.49	0.47	0.5	1 if taken loan in last 24 months and 0 otherwise
Non-agricultural income (D)	0.15	0.36	0.47	0.5	0.16	0.36	0.38	0.49	0.29	0.45	1 if HH has income from non-agriculture source, 0 otherwise
Farm labor income (D)	0.01	0.09	0.04	0.19	0.13	0.34	0.04	0.18	0.05	0.22	1 if works as on farm labor, 0 otherwise
Non-farm labor income (D)	0.03	0.18	0.25	0.43	0.19	0.38	0.15	0.34	0.15	0.36	1 if works as non-farm labor, 0 otherwise
Membership (D)	0.35	0.48	0.09	0.28	0.39	0.49	0.45	0.52	0.32	0.47	1 if any family member is member in any institution in village and 0 otherwise
Access to market and agriculture extension service, and training											
Distance to input market (C)	6.43	3.07	4.69	4.67	3.54	4.02	4.39	5.81	6.07	5.21	Distance to nearest input market from house (in km)
Distance to agriculture extension office (C)	5.25	2.84	5.04	3.66	9.16	8.79	3.72	3.56	6.78	6.80	Distance to agriculture extension service from house (in km)
Training (D)	0.22	0.41	0.52	0.5	0.04	0.18	0.02	0.15	0.2	0.4	1 if HH has received training on improved seeds, soil & water management, crop rotation, minimum tillage, 0 otherwise
Farmland characteristics											
Soil fertility (D)	0.91	0.23	0.46	0.50	0.24	0.35	0.37	0.49	0.48	0.39	1 if good and 0 otherwise
Soil depth (D)	0.38	0.19	0.15	0.36	0.42	0.49	0.09	0.38	0.27	0.41	1 if deep and 0 if shallow
Land slope (D)	0.91	0.29	0.47	0.50	0.75	0.43	0.56	0.47	0.68	0.46	1 if gentle slope and 0 if medium/steep slope
Irrigation (D)	0.99	0.07	0.92	0.17	0.76	0.46	0.56	0.38	0.75	0.45	1 if irrigated and 0 if rainfed
Soil salinity (D)	0.16	0.47	0.04	0.18	0.45	0.49	0.07	0.31	0.19	0.38	1 if soil salinity high and 0 if low
Land ownership (D)	0.88	0.33	0.75	0.43	0.62	0.48	0.89	0.32	0.78	0.37	1 if owner-operated plot and 0 if leased-in
Distance to plot (C)	1.39	0.99	0.76	1.06	0.59	0.82	0.87	1.15	0.81	0.83	Average distance from homestead to farm plot (in km)

1. C and D refer to continuous and dummy variables, respectively

2. na refers to not applicable

3. To capture the effect of wealth on the use of urea, DAP, and manure by the farm household, we constructed household asset index using principal component analysis (for detail, see https://www.stata.com/manuals13/mvpca.pdf). We included most of the household assets such as tractor, car, television, water pump, and motorbike for constructing household asset index

4. Adult equivalent

5. Tropical livestock unit (TLU): calculated using Chilonda, P., Otte, J., 2006. Indicators to monitor trends in livestock production at national, regional, and international levels. Livestock research for Rural Development 18. Article number 117. Accessed at http://www.lrrd.org/lrrd18/8/chil18117.htm

Unlike urea and DAP, farmers did not apply manure to all fields in the survey sample. In Bihar, (India) and Nepal, 47% of rice plots received manure, while 26% applied manure in Bangladesh. Manure was applied in 24% of plots cultivated to wheat in Nepal. Across all locations, farmers in focus groups indicated that their use of manure is decreasing over time. They reported that educated young household members are less interested in carrying manure to plots, and as a result, the use of chemical fertilizer is increasing over time. In Haryana, the average amounts of manure use in rice and wheat fields were 1,899 and 1,680 kg ha^−1^, respectively, while they were 925 and 1,250 kg ha^−1^ in rice and wheat fields in Bihar.

On the average, age of household heads ranges between 47 and 51 years. Nepal had the highest percentage of illiterate household heads (48%), while Haryana (India) has the lowest percentage of illiterate household heads (22%). Almost 38% of the sample households in Nepal had at least one member migrated for employment, while only 11% indicated out-migration from Haryana.

Average landholding size in Haryana was 3.33 ha, much greater than in other sites (0.44 to 0.51 ha). Livestock holdings in the study locations exhibited similar trends, with the highest in Haryana (3.81) followed by Nepal (1.25), Bangladesh (0.92), and Bihar (0.70). Access to credit is relatively better in Bangladesh (69%) compared with other locations (34–44%).

Farmland characteristics substantially vary across study sites. Farmers in Haryana self-described that almost 90% of their plots have fertile soil, whereas only 24% of farmers in Bangladesh suggested that their soil was fertile. Access to irrigation facilities varied considerably, with all farmers in Haryana indicating they could access irrigation, while 95% in Bihar, 56% in Nepal, and 76% in Bangladesh could access irrigation reliably.

### Factors influencing the amount of urea and DAP use in rice and wheat

Farmers’ application of urea and DAP are, in general, affected by similar factors (Table 3). Gender, education, and migration are key household characteristics significantly affecting the amount of urea applied in rice. Compared to female-headed households (FHHs), male-headed households (MHHs) in Bihar and Bangladesh applied significantly higher rates of urea in rice. However, MHHs in Nepal applied significantly less urea and DAP in rice compared to their FHH counterparts. It is expected, as Nepalese women face fewer restrictions in carrying out agricultural activities than in India or Bangladesh, where socio-cultural norms may inhibit them from doing so (Aryal et al. 2014; Mahmud et al. 2012; Mallick and Rafi 2010). Additionally, male out-migration has substantially changed the traditional gender norms in Nepal (Spangler and Christie 2020; Sugden et al. 2018). Compared to illiterate household heads, those with secondary and higher education also applied more urea and DAP to rice. This finding does not corroborate with Zhou et al. (2010) and Adesina (1996). Rural out-migration also appears to reduce fertilizer use in Bihar and Bangladesh, while it conversely increased fertilizer use in Nepal. Male out-migration negatively influenced fertilizer use in Bihar, India, and Bangladesh as fertilizer application is mainly the men’s task in these countries.

**Table 3:** Factors influencing amount of Urea (kg ha^−1^) and DAP (kg ha^−1^) used in rice in the study sites

	Urea (kg ha^**−**1^)				DAP (kg ha^**−**1^)			
	Haryana	Bihar	Bangladesh	Nepal	Haryana	Bihar	Bangladesh	Nepal
*Household (HH) characteristics*								
Male headed HH	19.76	22.17**	21.04***	−18.08***	4.04	8.12***	5.17***	−7.35***
	(21.36)	(11.02)	(7.06)	(6.07)	(6.56)	(2.14)	(1.95)	(2.33)
Age of HH head	0.32	4.81	1.47	3.31	0.73	−0.10	−0.37	−1.10
	(0.39)	(8.45)	(2.31)	(5.78)	(0.75)	(0.17)	(0.49)	(1.12)
Primary education	1.70	10.22*	7.63	9.70**	−4.56	3.61**	2.59	2.35***
	(9.26)	(5.41)	(8.12)	(4.33)	(5.83)	(1.67)	(1.83)	(0.81)
Secondary education	15.23***	13.72**	10.12**	18.87***	5.09**	3.63**	1.92**	2.04***
	(5.07)	(6.14)	(4.05)	(7.06)	(2.02)	(1.72)	(0.80)	(0.63)
Higher education	22.10**	28.75***	26.81***	20.35**	4.86***	3.97***	2.32***	2.83***
	(10.89)	(9.07)	(8.78)	(10.01)	(1.55)	(1.23)	(0.78)	(0.91)
Family size (AEC)	−20.49	−27.11	6.80**	17.49	−6.49	−5.51	2.53*	−7.88
	(39.23)	(28.71)	(3.05)	(16.74)	(14.40)	(8.11)	(1.40)	(8.01)
Migration	−11.29	−20.16***	−15.21**	25.08***	−6.19	−2.50**	−3.16**	4.21***
	(7.45)	(5.03)	(6.01)	(8.14)	(7.58)	(0.98)	(1.50)	(1.09)
*Economic and social capital*								
Land size	47.93***	22.15***	29.36***	25.11***	10.44***	7.21***	3.57**	5.40*
	(9.24)	(7.01)	(10.12)	(6.17)	(3.30)	(2.45)	(1.54)	(2.93)
Livestock owned	9.28**	5.46**	4.42**	4.04***	2.39	2.98***	4.13**	3.24***
	(4.01)	(2.24)	(2.00)	(1.14)	(1.92)	(1.03)	(2.01)	(1.21)
Asset index	5.34	4.17**	7.03***	2.82***	3.15	3.29**	3.10***	2.06**
	(6.76)	(1.98)	(2.59)	(0.76)	(3.31)	(1.31)	(1.09)	(0.83)
Credit access	−17.83	−6.74**	4.18***	5.74	−4.55	−3.12**	2.04***	−2.55
	(16.91)	(2.85)	(1.58)	(6.85)	(3.18)	(1.30)	(0.58)	(3.24)
Off-farm income	7.57***	12.48***	5.32**	11.81***	3.29**	3.54***	2.46***	2.53***
	(2.33)	(3.57)	(2.17)	(2.86)	(1.39)	(1.11)	(0.91)	(0.89)
Membership	4.84	5.08**	−18.84	4.84***	3.65	2.91**	5.14	2.63***
	(5.15)	(2.02)	(13.85)	(1.25)	(5.28)	(1.29)	(5.51)	(0.88)
*Access to market, extension service, and training*								
Distance to input market	−7.98***	−10.01***	−9.25***	−11.71***	−6.01***	−3.26**	−4.05***	−5.20***
	(2.78)	(3.04)	(2.37)	(3.14)	(1.35)	(1.50)	(0.98)	(1.04)
Distance to extension service	−0.80	−1.73	−3.48	−2.31	−0.60	−2.45	−0.87	−1.60
	(0.95)	(1.87)	(4.62)	(3.11)	(0.41)	(3.12)	(0.89)	(1.57)
Training	−3.53	5.61***	7.30**	5.02***	4.84**	6.16***	3.25***	2.93**
	(6.43)	(1.32)	(3.00)	(1.43)	(2.06)	(1.95)	(1.10)	(1.45)
*Farmland characteristics*								
Soil fertility	−10.10***	−15.21***	−12.44**	−9.07**	−4.22***	−2.83***	−5.72***	−6.01***
	(3.09)	(5.11)	(6.01)	(4.28)	(1.05)	(1.01)	(2.13)	(1.98)
Soil depth	−4.13	−12.07**	−10.39**	−9.22*	−3.35	−2.62***	−4.53***	−4.85***
	(7.07)	(5.14)	(4.72)	(5.41)	(3.27)	(0.97)	(1.27)	(1.40)
Land slope	5.33	9.53	7.81	6.72	4.21	3.35	2.18	3.41
	(10.62)	(9.21)	(8.18)	(8.29)	(4.85)	(4.07)	(3.66)	(3.30)
Irrigation	na	29.54	39.12***	36.41***	na	8.71	13.95***	11.85***
	na	(33.45)	(12.37)	(11.83)	na	(8.60)	(3.77)	(2.91)
Soil salinity	10.15***	5.15	15.39***	4.15	7.99**	−3.99	5.84***	−2.75
	(2.49)	(7.19)	(5.12)	(6.14)	(3.41)	(3.63)	(2.05)	(3.17)
Land ownership	15.10**	22.01***	27.41***	18.05*	10.22**	12.83***	9.01***	9.42***
	(6.44)	(7.09)	(9.07)	(10.01)	(5.05)	(4.10)	(3.04)	(2.81)
Distance to plot	−11.95***	−18.07***	−17.58***	−21.17***	−7.85***	−9.25***	−8.53***	−7.15***
	(3.08)	(5.14)	(5.13)	(5.06)	(1.53)	(2.04)	(2.17)	(1.91)
Amount of urea applied	na	na	na	na	0.19***	0.13***	0.07**	0.09***
	na	na	na	na	(0.05)	(0.04)	(0.03)	(0.03)
Amount of DAP applied	0.55***	0.46***	0.17***	0.21***	na	na	na	na
	(0.17)	(0.11)	(0.03)	(0.05)	na	na	na	na
Constant	143.45***	115.37	122.21***	107.08***	89.34***	67.90***	46.15***	43.26***
	(31.41)	(20.24)	(24.04)	(29.37)	(22.21)	(19.40)	(11.65)	(11.33)
R-squared	0.53	0.59	0.47	0.64	0.61	0.68	0.51	0.66
Number of observations	667	1299	1182	1576	667	1299	1182	1576

Note: Standard errors in parenthesis. *, **, and ** refer to 10%, 5%, and 1% level of significance, respectively. “na” refers to not applicable

Farm size was positively associated with the amount of both urea and DAP applied in rice. Households with more livestock and wealthier households (measured in terms of AI) in all study locations except Haryana have applied more urea and DAP. Access to credit is positively associated with the application of urea and DAP in rice in Bangladesh, while the reverse was found in Bihar. Off-farm income, market access, and training positively influenced the application of urea and DAP across all locations. These findings suggest that it may be preferential for government policy to focus on facilitating additional opportunities for off-farm employment and income generation, increasing market access, and training rather than on access to credit.

Farmers applied less urea and DAP to fields they indicated were more inherently fertile in comparison to those they deemed as less fertile. Irrigation was positively associated with fertilizer rates in Bangladesh and Nepal, but not in Bihar. Farmers with secured tenure also tended to apply higher rates of fertilizer compared to those who rented land. Most farmers are found to use urea and DAP as complementary inputs in rice farming. We also observed that factors affecting the amount of urea and DAP applied to wheat (Table 4) are generally similar to that of rice in all countries (see Table 3).

**Table 4:** Factors influencing the amount of urea (kg ha^−1^) and DAP (kg ha^−1^) used in wheat in the study sites

		Urea (kg ha^−1^)		DAP (kg ha^−1^)		
	Haryana	Bihar	Nepal	Haryana	Bihar	Nepal
*Household (HH) characteristics*						
Male headed HH	22.68	18.09**	−21.32***	7.12	10.08***	−5.18**
	(25.45)	(9.10)	(7.12)	(7.23)	(2.81)	(2.50)
Age of HH head	0.46	6.75	5.24	0.93	−0.85	−2.13
	(0.51)	(7.04)	(4.91)	(0.92)	(0.94)	(2.10)
Primary education	3.67	9.36***	11.51**	−6.11	3.01***	3.35***
	(5.92)	(3.21)	(5.24)	(6.07)	(1.02)	(0.96)
Secondary education	12.17**	17.23***	15.46***	7.10**	4.52**	3.12***
	(6.03)	(4.10)	(5.13)	(3.31)	(2.03)	(0.89)
Higher education	24.08***	19.14***	18.19***	5.14***	4.61***	2.96***
	(7.02)	(5.17)	(6.42)	(1.28)	(1.50)	(0.97)
Family size (AEC)	−15.94	−13.28	−22.47	−11.46	−7.63	−8.78
	(16.11)	(12.97)	(23.19)	(13.22)	(8.92)	(8.25)
Migration	−13.42	−24.20***	19.25***	−7.16	−4.54**	7.23***
	(9.73)	(4.31)	(5.80)	(7.95)	(1.25)	(2.42)
*Economic and social capital*						
Land size	25.24***	27.08***	19.50***	12.40***	9.20***	6.71***
	(7.51)	(7.74)	(5.42)	(4.16)	(3.15)	(2.09)
Livestock owned	11.14***	8.79***	6.58***	3.56***	5.89***	5.20***
	(3.89)	(3.04)	(2.01)	(0.98)	(1.81)	(1.43)
Asset index	7.85	7.50***	3.51**	4.69	5.23***	2.21***
	(7.76)	(2.10)	(1.72)	(4.37)	(1.31)	(0.84)
Credit access	−12.33	−4.28*	4.08	−3.15	−4.12***	−3.57
	(13.61)	(2.55)	(3.85)	(3.45)	(1.47)	(3.33)
Off-farm income	11.10***	8.92***	15.01***	6.21***	7.11***	5.63***
	(3.30)	(2.54)	(3.69)	(2.08)	(2.45)	(1.39)
Membership	5.73	4.25**	5.91***	5.19	4.81**	3.95***
	(5.44)	(1.97)	(1.30)	(5.13)	(1.88)	(0.98)
Distance to input market	−10.77***	−17.15***	−14.50***	−7.01***	−8.53**	−10.24***
	(3.14)	(4.10)	(3.36)	(2.30)	(2.57)	(3.15)
Distance to extension service	−2.96	−3.27	−1.67	−0.96	−3.45	−2.93
	(3.98)	(4.13)	(2.56)	(0.94)	(3.32)	(2.87)
Training	8.34**	3.96***	7.02***	5.04**	7.06***	4.90**
	(3.63)	(1.25)	(2.08)	(2.25)	(2.14)	(1.85)
*Farmland characteristics*						
Soil fertility	−17.51***	−12.42***	−11.90***	−7.20***	−5.08***	−6.78***
	(5.11)	(3.28)	(3.59)	(2.12)	(1.39)	(1.68)
Soil depth	−5.09*	−9.21***	−7.50***	−4.89	−4.42***	−5.05***
	(2.68)	(3.10)	(2.41)	(4.73)	(1.06)	(1.53)
Land slope	7.08	8.23	4.65	3.80	3.89	4.74
	(9.60)	(8.44)	(6.88)	(4.71)	(4.11)	(5.30)
Irrigation	na	21.59	30.20***	na	9.15	13.50***
	na	(24.32)	(8.73)	na	(10.01)	(3.21)
Soil salinity	15.05***	6.86	6.33	8.50**	−4.74	−2.95
	(3.61)	(7.04)	(6.44)	(3.56)	(4.63)	(3.47)
Land ownership	14.50**	25.27***	12.01**	9.02**	13.33***	10.14***
	(5.69)	(6.25)	(5.21)	(4.15)	(3.85)	(2.98)
Distance to plot	−10.04***	−20.55***	−23.50***	−6.81***	−10.92***	−9.55***
	(3.10)	(5.25)	(6.01)	(1.61)	(2.48)	(2.01)
Amount of urea applied	na	na	na	0.17***	0.11***	0.15***
	na	na	na	(0.04)	(0.03)	(0.03)
Amount of DAP applied	0.61**	0.45***	0.28***	na	na	na
	(0.19)	(0.13)	(0.10)	na	na	na
Constant	155.23***	121.33***	113.04***	71.35***	63.14***	47.26***
	(29.87)	(22.19)	(24.97)	(16.41)	(15.08)	(12.37)
R-squared	0.57	0.59	0.45	0.65	0.48	0.62
Number of observations	667	1544	977	667	1544	977

Note: Standard errors in parenthesis. *, **, and ** refer to 10%, 5%, and 1% level of significance, respectively. “na” refers to not applicable

### Factors determining the adoption and intensity of manure use in rice and wheat

Wald chi-square tests for the Heckman two-step models were significant at 1% level (Tables 5 and 6), indicating the validity of the models in explaining observed differences in farmers’ decision to apply manure in rice and wheat. The Inverse Mills Ratio was highly significant in all models, implying that manure use intensity depends on the likelihood of farmers to decide to apply manure. Sets of the observed factors appear to affect the likelihood of farmers’ choice to utilize manure. These factors differed from the ways they influence the rates of manure used among the subset of farmers who chose to apply manure. Furthermore, some factors appear to have contradictory effects on the choice to apply manure vis-à-vis its application rate. As several factors influencing the adoption and intensity of adoption of manure in rice (Table 5) and wheat (Table 6) are similar, we describe their results together.

**Table 5:** Factors influencing adoption and amount of manure used in rice in the study sites (Heckman two-step model)

	Haryana		Bihar		Bangladesh		Nepal	
Variables	Adoption	Intensity	Adoption	Intensity	Adoption	Intensity	Adoption	Intensity
*Household (HH) characteristics*								
Male headed HH	0.09	−271.19	0.40**	−351.33	0.29**	151.51**	0.35**	220.22**
	(0.17)	(305.04)	(0.18)	(335.99)	(0.13)	(61.33)	(0.14)	(105.10)
Age of HH head	−0.03	21.51	−0.01*	11.15	−0.09***	9.37	−0.10**	8.14
	(0.13)	(18.81)	(0.00)	(7.79)	(0.03)	(10.70)	(0.04)	(8.31)
Primary education	0.28***	134.17	0.46***	155.71	0.34***	234.18	0.38***	184.16
	(0.10)	(213.04)	(0.14)	(316.02)	(0.11)	(242.02)	(0.09)	(213.10)
Secondary education	−0.19**	264.56**	−0.23**	364.55**	−0.21***	205.17**	−0.31**	195.22**
	(0.09)	(112.15)	(0.10)	(175.15)	(0.07)	(95.42)	(0.15)	(83.14)
Higher education	−0.12***	298.16***	−0.12	304.79**	−0.12	271.83**	−0.12	276.79**
	(0.03)	(100.21)	(0.13)	(152.31)	(0.13)	(135.30)	(0.13)	(122.26)
Family size (AEC)	0.39***	109.58	0.36***	153.28**	0.25***	201.58**	0.41***	185.58**
	(0.13)	(256.75)	(0.10)	(76.75)	(0.09)	(96.75)	(0.11)	(84.23)
Migration	0.04	103.70	−0.16*	−203.70**	−0.13**	−214.08**	−0.19**	−195.03**
	(0.07)	(122.95)	(0.09)	(89.23)	(0.06)	(101.11)	(0.09)	(91.12)
*Economic and social capital*								
Land size	0.23***	−201.09	0.43***	−199.37*	0.51***	−205.51**	0.39***	−185.72**
	(0.06)	(230.22)	(0.08)	(112.30)	(0.17)	(97.30)	(0.12)	(75.14)
Livestock owned	0.53***	289.01**	0.31***	270.19***	0.25***	270.15***	0.35***	300.09***
	(0.06)	(124.71)	(0.10)	(101.11)	(0.04)	(94.17)	(0.07)	(99.38)
Asset index	−0.09	35.33	0.16**	235.33**	0.27***	151.74**	0.21**	135.33**
	(0.08)	(134.92)	(0.07)	(104.27)	(0.09)	(71.32)	(0.10)	(61.92)
Credit access	−0.07	130.08**	−0.15*	290.86*	0.22**	150.54**	0.19***	175.80***
	(0.13)	(63.51)	(0.09)	(166.54)	(0.10)	(69.14)	(0.07)	(59.66)
Off-farm income	0.42***	−182.47	0.47***	−171.41	0.33***	−166.78	0.27***	−153.77
	(0.12)	(206.32)	(0.14)	(226.13)	(0.10)	(168.23)	(0.09)	(154.09)
Membership	0.23*	−150.41	0.35***	−189.24	0.47***	−171.41	0.19***	−187.02
	(0.11)	(186.22)	(0.09)	(226.13)	(0.14)	(189.01)	(0.06)	(211.26)
*Access to market, extension service and training*								
Distance to input market	0.06***	16.63	0.10***	125.16***	0.09***	161.07***	0.13***	152.60**
	(0.01)	(27.52)	(0.03)	(39.27)	(0.03)	(51.27)	(0.04)	(67.15)
Distance to extension service	0.01	−20.27	0.13	−19.60	0.08	−22.03	0.12	−33.10
	(0.01)	(21.56)	(0.18)	(20.41)	(0.10)	(25.01)	(0.21)	(35.25)
Training	0.18*	146.28	0.22**	164.82**	0.15**	146.28**	0.25**	154.83**
	(0.10)	(154.60)	(0.09)	(79.45)	(0.07)	(70.60)	(0.10)	(72.01)
*Farm land characteristics*								
Soil fertility	0.58***	−314.12**	0.26***	−331.01*	0.34***	−290.13**	0.29***	−243.31**
	(0.08)	(135.27)	(0.06)	(185.21)	(0.11)	(144.45)	(0.09)	(109.21)
Soil depth	0.60***	−442.80	0.53**	−272.39	0.41***	−248.45	0.38***	−215.44
	(0.13)	(283.92)	(0.23)	(271.83)	(0.11)	(239.97)	(0.10)	(220.02)
Land slope	0.01	−37.49*	0.07	−82.14***	0.21	−67.49**	0.14	−41.49*
	(0.01)	(22.73)	(0.09)	(19.22)	(0.22)	(31.73)	(0.13)	(22.73)
Irrigation	na	na	−0.38	145.01	0.37***	318.01***	0.41***	250.01**
	na	na	(0.41)	(151.50)	(0.12)	(110.50)	(0.09)	(115.27)
Soil salinity	−0.39***	−319.02***	−0.40***	−223.13**	−0.34***	−285.45**	−0.25***	−201.23**
	(0.11)	(87.32)	(0.13)	(102.97)	(0.10)	(112.28)	(0.09)	(98.43)
Land ownership	0.61***	396.09**	0.49***	301.25***	0.42***	250.89***	0.47***	199.15**
	(0.20)	(175.41)	(0.16)	(101.01)	(0.13)	(96.28)	(0.15)	(91.14)
Distance to plot	−0.38***	−459.18**	−0.27***	−350.51***	−0.31***	−259.02**	−0.41***	−249.05**
	(0.09)	(182.21)	(0.08)	(98.10)	(0.10)	(110.48)	(0.12)	(102.48)
Constant	4.73***	536.45***	5.44***	442.36***	3.46***	336.21**	4.91***	350.35**
	(1.69)	(164.23)	(2.10)	(102.62)	(1.25)	(164.29)	(1.82)	(155.71)
Mills lambda		−0.073***		−0.092***		−0.107***		−0.113***
		(0.017)		(0.023)		(0.041)		(0.037)
Wald Chi-square (23)	178.39***		168.83***		193.21***		159.11***	
Prob >chi2	0.000		0.000		0.000		0.000	
Number of observations (plots)	665	306	1299	611	1182	307	1576	740

Note: Standard errors in parenthesis. *, **, and ** refer to 10%, 5%, and 1% level of significance, respectively. “na” refers to not applicable

**Table 6:** Factors influencing adoption and amount of manure used in wheat in the study sites (Heckman two-step model)

	Haryana		Bihar		Nepal	
Variables	Adoption	Intensity	Adoption	Intensity	Adoption	Intensity
*Household (HH) characteristics*						
Male headed HH	−0.31	−254.21	0.27**	−271.51	0.24***	241.27**
	(0.33)	(295.41)	(0.11)	(319.13)	(0.09)	(112.05)
Age of HH head	−0.05	19.15	−0.06**	23.45	−0.13***	11.21
	(0.17)	(20.63)	(0.03)	(24.12)	(0.05)	(11.34)
Primary education	0.22**	173.24	0.37***	185.55	0.41***	199.68
	(0.09)	(203.13)	(0.11)	(301.16)	(0.11)	(201.31)
Secondary education	−0.23**	312.21***	−0.27**	293.55**	−0.19	201.91**
	(0.09)	(117.25)	(0.13)	(145.15)	(0.09)	(97.38)
Higher education	−0.19***	268.17***	−0.12	271.04*	−0.17	209.73**
	(0.06)	(98.61)	(0.14)	(141.31)	(0.13)	(101.06)
Family size (AEC)	0.27***	153.51*	0.29***	161.83**	0.32***	192.24**
	(0.08)	(79.15)	(0.10)	(72.66)	(0.11)	(91.22)
Migration	0.01	−116.12	−0.22***	−220.24**	0.12**	−205.11**
	(0.03)	(129.28)	(0.08)	(101.01)	(0.05)	(97.27)
*Economic and social capital*						
Land size	0.25***	−260.17*	0.39***	−205.31**	0.41***	−198.28***
	(0.07)	(156.21)	(0.12)	(97.33)	(0.14)	(71.14)
Livestock owned	0.49***	277.14**	0.38***	255.51***	0.29***	271.15***
	(0.11)	(120.71)	(0.12)	(85.22)	(0.07)	(95.39)
Asset index	−0.09	75.56	0.17***	254.11**	0.19**	122.30**
	(0.10)	(113.34)	(0.05)	(123.27)	(0.09)	(60.29)
Credit access	−0.12	110.08**	−0.21***	274.07*	0.21***	181.07***
	(0.13)	(51.05)	(0.08)	(150.04)	(0.07)	(60.16)
Off-farm income	0.37***	−195.02	0.42***	−198.01	0.31***	−157.54
	(0.12)	(198.06)	(0.13)	(207.26)	(0.10)	(160.04)
Membership	0.31**	−171.28	0.27***	−199.18	0.24***	−192.31
	(0.15)	(179.20)	(0.09)	(208.81)	(0.06)	(198.61)
*Access to market, extension service, and training*						
Distance to input market	0.09***	28.25	0.13***	105.45***	0.17***	141.09**
	(0.02)	(29.04)	(0.04)	(31.09)	(0.04)	(65.07)
Distance to extension service	0.07	−42.11	0.21	−25.14	0.12	−33.10
	(0.09)	(51.50)	(0.23)	(27.43)	(0.21)	(35.25)
Training	0.22**	153.42	0.25***	150.02**	0.33**	142.83**
	(0.10)	(156.04)	(0.07)	(70.41)	(0.11)	(64.07)
*Farmland characteristics*						
Soil fertility	0.37***	−300.18***	0.18***	−250.01*	0.26***	−261.34**
	(0.11)	(110.02)	(0.06)	(146.01)	(0.08)	(109.11)
Soil depth	0.45***	−275.17	0.43**	−251.41	0.32***	−198.15
	(0.13)	(278.08)	(0.19)	(259.17)	(0.10)	(201.04)
Land slope	0.07	−60.41**	0.10	−76.06***	0.11	−46.09**
	(0.06)	(30.05)	(0.09)	(24.91)	(0.13)	(22.73)
Irrigation	na	na	−0.40	152.09	0.27***	271.05**
	na	na	(0.43)	(157.50)	(0.09)	(130.17)
Soil salinity	−0.41***	−295.42***	−0.24***	−210.74**	−0.27***	−210.31**
	(0.12)	(81.32)	(0.08)	(100.97)	(0.09)	(97.44)
Land ownership	0.58***	291.09**	0.39***	285.09***	0.42***	201.36**
	(0.19)	(146.41)	(0.13)	(97.22)	(0.14)	(99.18)
Distance to plot	−0.27***	−350.18***	−0.29***	−311.01***	−0.33***	−260.14***
	(0.09)	(120.21)	(0.10)	(94.21)	(0.08)	(91.08)
Constant	5.29***	610.81***	3.98**	421.14***	4.85***	371.61***
	(1.74)	(200.63)	(1.81)	(141.01)	(1.62)	(141.71)
Mills lambda		−0.044***		−0.051***		−0.321***
		(0.007)		(0.010)		(0.102)
Wald Chi-square (23)	173.21***		167.96***		158.54***	
Prob > chi2	0.000		0.000		0.000	
Number of observations (plots)	667	167	1544	689	977	235

Note: Standard errors in parenthesis. *, **, and ** refer to 10%, 5%, and 1% level of significance, respectively. “na” refers to not applicable

Compared to FHHs, MHHs in Bihar, Bangladesh, and Nepal appear to be more likely to choose to use manure, but the rates of application are higher among MHHs in Bangladesh and Nepal only. Farmers’ educational levels have differential impacts on the likelihood to use and rate of application. Household heads with primary education (relative to no formal schooling) were more likely to apply manure, though the same variable failed to influence the amount of manure applied. Household heads with secondary and higher educational levels were conversely less likely to apply manure. However, if they do apply manure, the rates of use were higher than those with no or primary education only.

The likelihood of manure application increased with land size, but the rate of application was inversely related. Farmers reported that the young and educated generation tend not to prefer labor-intensive approaches such as manure application. Livestock ownership, the major source of manure, was unsurprisingly positively associated with both the likelihood and intensity of manure use in all sites. Access to credit had variable effects on manure use: no impact was observed in Haryana, negative in Bihar, and positive in Bangladesh and Nepal. Factors such as off-farm income, market access, training, and membership in village institutions also increased manure use in both crops.

Farmers are also less likely to apply manure if they perceive the land is highly saline. Use of organic amendments has however, been shown to help mitigate the effects of salinity on crop productivity (Ding et al. 2020; Xu et al. 2014). Educational programs could be of use in saline-affected locations where farmers have access to manure and can afford labor to apply it, although application over multiple seasons may be required for desirable impacts (Ding et al. 2020). Those who did apply manure also used lower rates. Similar is the findings for plots that are located farther from the homestead. By contrast, farmers were more likely to adopt manure in fields they perceived of having greater soil depth. In Bangladesh and Nepal, irrigation was also positively associated with both the likelihood and rate of manure use. Compared to rented fields, farmers with tenure also had greater rates of manure application.

### Factors affecting the over-use of N fertilizer

A total of 33.6% of rice fields in our data were reported by farmers as receiving more than 120 kg N ha^−1^, and 18.5% of the wheat fields received more than 140 kg N ha^−1^ (see Appendix: Tables 8, 9 and 10). Though the critical value of N that is used here for defining over-use of N may vary across sites, we believe it to be rational here as our objective is to examine the factors contributing to the over-use of N. Moreover, we estimated the models by changing the critical value of N for rice as more than 100 kg N ha^−1^ and for wheat 120 kg N ha^−1^, the variables significantly affecting the over-use of N in rice and wheat remained almost the same. Therefore, we analyzed the factors affecting the over-use of fertilizer by combining data from all locations (Table 7).

**Table 7:** Factors influencing the over-use of N fertilizer (kg ha^−1^) in rice and wheat

Variables	Rice	Wheat
*Household (HH) characteristics*		
Male headed HH	0.30**	0.39***
	(0.15)	(0.14)
Age of HH head	0.02	0.00
	(0.03)	(0.00)
Primary education (base category: illiterate)	0.11	0.13
	(0.19)	(0.21)
Secondary education (base category: illiterate)	0.12***	0.10**
	(0.04)	(0.05)
Higher education (base category: illiterate)	0.31***	0.27**
	(0.09)	(0.11)
Family size (AEC)	0.16**	0.29
	(0.07)	(0.31)
Migration	0.07	0.10
	(0.08)	(0.11)
*Economic and social capital*		
Land size	0.28***	0.31***
	(0.10)	(0.09)
Livestock owned	0.09**	0.15***
	(0.04)	(0.05)
Asset index	0.17***	0.12**
	(0.05)	(0.6)
Credit access	−0.22	0.18
	(0.23)	(0.20)
Off-farm income	0.09***	0.07***
	(0.03)	(0.02)
Membership	0.13	0.10**
	(0.12)	(0.05)
*Access to market, extension service, and training*		
Distance to input market	−0.11***	−0.13***
	(0.03)	(0.04)
Distance to extension service	0.06	0.11
	(0.07)	(0.13)
Training on fertilizer management	−0.11***	−0.12***
	(0.03)	(0.04)
*Farmland characteristics*		
Soil fertility	−0.11***	−0.08***
	(0.03)	(0.02)
Soil depth	−0.13**	−0.15***
	(0.05)	(0.03)
Land slope	−0.11	0.14
	(0.12)	(0.16)
Irrigation	0.46***	0.39***
	(0.15)	(0.13)
Soil salinity	−0.09	−0.13
	(0.08)	(0.12)
Land ownership	0.17***	0.21***
	(0.05)	(0.06)
Distance to plot	−0.12***	−0.06***
	(0.04)	(0.02)
*Study locations dummy*		
Haryana (base category: Nepal)	0.39***	0.35***
	(0.10)	(0.11)
Bihar (base category: Nepal)	0.18***	0.21***
	(0.06)	(0.07)
Bangladesh (base category: Nepal)	0.29***	na
	(0.09)	na
Constant	−3.11***	−2.13***
	(0.63)	(0.55)
LR chi (26)	173.62	163.78
Prob > chi2	0.000	0.000
Pseudo R-Squared	0.23	0.20
Censored observations	3777	2675
Uncensored observations	937	513
Total number of observations	4712	3188

Note: Standard errors in parenthesis. *, **, and ** refer to 10%, 5%, and 1% level of significance, respectively. “na” refers to not applicable

Compared to FHHs, MHHs appear to be more likely to over-use N. Household heads with secondary and higher education levels also appear to have a higher likelihood to over-use of N (significant at 5% and 1% level). Land size, off-farm income, and wealth were also significantly and positively associated with the over-use of N.

Households that are farther from the market are perhaps unsurprisingly less likely to over-use N fertilizer. Training on fertilizer management is negatively associated with the N over-use. Farmers are not likely to over-use N fertilizer in plots they consider to have higher inherent fertility levels or have deeper soil profiles. Over-use of N fertilizer does appear to increase with access to irrigation, consistent findings as observed in the states with successful GR. Across the study locations, Nepal has the lowest percentage of over-use of N in both rice and wheat. All other countries conversely have a greater likelihood of overusing N.

Overall, a wide variation is observed across the study sites regarding the over-use of N fertilizer in rice and wheat. The findings indicate that training on fertilizer management is more crucial than formal education to reduce the over-use of N. This also calls for a new study focusing on the institutional analysis, including the information and support that farmers get from extension agents to regularly update the fertilizer need for their farm, information on soil testing and nutrient adjustment as per requirement, and increased access to nutrient management tools.

### Farmer focus group findings

Our discussions with farmers in the study villages confirmed the complex interactions of variables that condition organic and inorganic fertilizer use decisions. Many farmers consider that application of organic fertilizer (manure) is very crucial for long-term soil fertility enhancement. However, they have no idea of the potential negative environmental externalities of N leaching from organic fertilizer. Although many farmers indicated that they could not clearly understand or “separate” the impact of a single production factor such as fertilizer to distill its implication for overall crop productivity, they did, however, clearly indicate that they understand the importance of fertilizer but lack knowledge on its balanced application. Therefore, they are more likely to go for over-application of N, mainly from urea, ignoring other nutrients.

Farmers reported that government subsidy plays an important role in fertilizer use. In all South Asian countries exempting Sri Lanka, government subsidy is higher on urea than on other fertilizers. In focus groups, farmers indicated that this has a strong influence on their application of fertilizer N at rates that are often higher than required. Together, the uneven subsidy structure also works as disincentives in using recommended rates of secondary and micronutrients. This is in line with the findings of Sapkota et al. (2018a).

Farmers also reported that an increase in formal education level may not necessarily reduce the over-use of N fertilizer as more educated family members have more non-farm opportunities and may not stay as involved in agriculture. Therefore, agricultural training, awareness raising, appropriate extension services – which can be delivered by the public and also by the private sector – and interventions to increase farmers’ knowledge of nutrient responses to plant are important to encourage change in farmers’ knowledge and behavior toward fertilizer use to reduce inefficiencies (Afrad et al. 2019; Ananth et al. 2019; Sapkota et al. 2018a). Some farmers also reported that traditional extension service by government institutions is not very effective in providing recent advancements in fertilizer management properly, and thus, there is a need to mobilize local youth clubs to update the recent developments in soil fertility management.

Very few participants were aware of the possibility of non-point water pollution due to nitrogen leaching from the over-use of N and its negative human health and environmental impacts. This underscores the importance of programs to educate farmers on the principles of efficient fertilizer management – perhaps coupled with appropriate nutrient management policy (Kishore et al. 2019).

## Conclusions and policy implications

This study revealed that various factors influence the application of inorganic (urea and DAP) and organic fertilizers (manure) in rice and wheat in South Asia. Wealth, gender, education, migration, access to market, training, and off-farm income sources are some of the key factors influencing the application of organic and inorganic fertilizers. Economic and elements of social capital are primarily positively correlated with increased inorganic fertilizer use. Discussions with farmers show that they prefer using off-farm income to purchase agricultural inputs rather than credit. Thus, public policy that creates off-farm income generation opportunities are likely to be of use – particularly for resource-poor and smallholder farm families – when increasing fertilizer use are the goal. Conversely, where farmers routinely over-apply fertilizers, or practice imbalanced application, more complex policy, and development interventions may be needed, including educational programs, direct training, and behavioral nudging methods that may encourage more rational use. Similarly, few farmers in the study locations appeared to have sophisticated knowledge on the negative effects of fertilizer over-use. Thus, training on fertilizer management, along with the dissemination of knowledge on negative externalities of its inappropriate use is likely to be important in preventing unsound over-use.

Another crucial finding is that application of manure is decreasing in all locations. Perhaps surprisingly, where farmers have higher levels of education, our data suggest that they are more likely to opt-out of manure application to rice and wheat fields. Combined with evidence on decreasing concentrations of organic matter in our study countries, this is arguably concerning and indicates the need for appropriate educational and extension programs, methods to offset the high labor demand, and costs of manure application, possibly through scale-appropriate machinery options to encourage educated and young farmers to use manure when and where it is most needed to maintain long-term soil fertility.

## Appendix

**Table 8:** Average N fertilizer applied in rice (in kg ha^−1^)

Study sites	Urea (A)	DAP (B)	Manure		Total N	
			0.5%N (C)	2%N(D)	A+B+C	A+B+D
Haryana	144.9	23	9.51	37.98	177.4	205.88
Bihar	96.6	17.1	4.625	18.5	118.3	132.2
Nepal	94.3	11.6	6.81	27.24	112.3	133.14
Bangladesh	131.1	11.7	5.6	22.4	148.4	165.2

**Table 9:** Average N fertilizer applied in wheat (in kg ha^−1^)

Study sites	Urea (A)	DAP (B)	Manure		Total N	
			0.5%N(C)	2%N(D)	A+B+C	A+B+D
Haryana	147.2	22.5	8.4	33.6	178.1	203.3
Bihar	103.5	19.8	6.25	25	129.6	148.3
Nepal	96.6	14.22	5.15	20.6	115.9	131.4

**Table 10:** Factors influencing the over-use of N from inorganic fertilzer only (kg ha^−1^) in rice and wheat

Variables	Rice	Wheat
*Household (HH) characteristics*		
Male headed HH	0.34*	0.41**
	(0.18)	(0.17)
Age of HH head	0.01	0.00
	(0.03)	(0.00)
Primary education (base category: illiterate)	0.06	0.09
	(0.13)	(0.11)
Secondary education (base category: illiterate)	0.09***	0.17**
	(0.02)	(0.09)
Higher education (base category: illiterate)	0.22**	0.18**
	(0.10)	(0.08)
Family size (AEC)	0.32	0.29
	(0.38)	(0.31)
Migration	0.07	0.10
	(0.08)	(0.11)
*Economic and social capital*		
Land size	0.31***	0.42***
	(0.14)	(0.09)
Livestock owned	−0.15***	−0.12***
	(0.03)	(0.04)
Asset index	0.13***	0.11**
	(0.04)	(0.5)
Credit access	−0.12	−0.09
	(0.08)	(0.13)
Off-farm income	0.09***	0.06***
	(0.03)	(0.02)
Membership	0.01	0.11**
	(0.01)	(0.05)
*Access to market, extension service, and training*		
Distance to input market	−0.16***	−0.19***
	(0.05)	(0.03)
Distance to extension service	0.05	0.10
	(0.07)	(0.13)
Training on fertilizer management	−0.07***	−0.09***
	(0.02)	(0.03)
*Farmland characteristics*		
Soil fertility	−0.08***	−0.07***
	(0.03)	(0.02)
Soil depth	−0.12**	−0.15***
	(0.05)	(0.03)
Land slope	−0.15	0.12
	(0.12)	(0.16)
Irrigation	0.52***	0.59***
	(0.15)	(0.14)
Soil salinity	−0.10	−0.11
	(0.08)	(0.12)
Land ownership	0.21***	0.18***
	(0.05)	(0.06)
Distance to plot	−0.11***	−0.07***
	(0.03)	(0.02)
*Study locations dummy*		
Haryana (base category: Nepal)	0.43***	0.37***
	(0.11)	(0.13)
Bihar (base category: Nepal)	0.21***	0.24***
	(0.07)	(0.06)
Bangladesh (base category: Nepal)	0.29***	na
	(0.11)	na
Constant	−2.09***	−1.85***
	(0.67)	(0.58)
LR chi (26)	161.21	183.63
Prob > chi2	0.000	0.000
Pseudo R-squared	0.21	0.17
Censored observations	3777	2675
Uncensored observations	937	513
Total number of observations	4712	3188

Note: Standard errors in parenthesis. *, **, and ** refer to 10%, 5%, and 1% level of significance, respectively. “na” refers to not applicable

## Footnotes

1 https://sustainabledevelopment.un.org/
